# Acute cannabidiol administration reduces alcohol craving and cue-induced nucleus accumbens activation in individuals with alcohol use disorder: the double-blind randomized controlled ICONIC trial

**DOI:** 10.1038/s41380-024-02869-y

**Published:** 2024-12-12

**Authors:** Sina Zimmermann, Anton Teetzmann, Joscha Baeßler, Lena Schreckenberger, Judith Zaiser, Marlen Pfisterer, Manuel Stenger, Patrick Bach

**Affiliations:** 1 Department of Addictive Behavior and Addiction Medicine, Central Institute of Mental Health, Medical Faculty Mannheim/ University of Heidelberg, Heidelberg, Germany; 2 German Center for Mental Health (DZPG), Mannheim, Germany; 3 Feuerlein Center on Translational Addiction Medicine (FCTS), University of Heidelberg, Heidelberg, Germany; 4 Department of Clinical Neuroscience, Karolinska Institutet, Stockholm, Sweden

**Keywords:** Addiction, Neuroscience, Drug discovery

## Abstract

Although alcohol use disorder (AUD) is highly prevalent, only a few medications are approved for its treatment leaving much room for improvement. Cannabidiol (CBD) might be a particularly promising candidate, with preclinical data suggesting that CBD is effective in targeting AUD symptoms and disease processes that drive alcohol use and relapse, due to its anti-craving, stress-reducing, and anti-compulsive effects. Here we report data from the double-blind randomized controlled ICONIC trial that compared the effects of a single dose of 800 mg cannabidiol against placebo (PLC) in N = 28 individuals with AUD. Cue-induced nucleus accumbens (NAc) activation, alcohol craving during a combined stress- and alcohol cue exposure session, as well as craving during an fMRI alcohol cue-reactivity task and CBD plasma levels served as outcomes. Individuals receiving CBD showed lower bilateral cue-induced NAc activation (*t*_*left*_NAc(23)_ = 4.906, *p* < 0.001, d = 1.15; *t*_right_NAc (23)_ = 4.873, *p* < 0.001, d = 1.13) and reported significantly lower alcohol craving after a combined stress- and alcohol cue exposure session (*F*_group(1,26)_ = 4.516, *p* = 0.043, eta^2^ = 0.15) and during the fMRI cue-reactivity task (*F*_group(1,24)_ = 6.665, *p* = 0.015, eta^2^ = 0.23). CBD levels were significantly higher in the CBD group (*t*_(25)_ = 3.808, *p* < 0.001, d = 1.47) and showed a significant negative association with alcohol craving during the cue exposure experiment (*r* = −0.394, *p*_FDR_ = 0.030) and during fMRI (*r* = −0.389, *p*_FDR_ = 0.030), and with left and right NAc activation (*r*_left___NAc_ = −0.459, *p*_FDR_ = 0.030; *r*_right___NAc_ = −0.405, *p*_FDR_ = 0.030). CBD’s capacity to reduce stress- and cue-induced alcohol craving and to normalize NAc activation – a region critical to the pathophysiology of AUD – contribute to understanding the neurobiological basis of its clinical effects and support its potential as a treatment option for AUD. Clinical Trials Registry: DRKS00029993.

## Introduction

Alcohol use disorder (AUD) is one of the most prevalent and devastating diseases globally [1]. Currently, the majority of AUD patients relapse even if treated with pharmacological relapse-preventive medication, stressing the need for developing new pharmacological treatments [2]. Cannabidiol (CBD) might be a promising candidate, due to its effects on substance use and craving. With regards to the specific effects of CBD on alcohol consumption, preclinical studies demonstrated that administration of CBD reduces the reinforcing properties of alcohol and decreases cue- and stress-induced alcohol self-administration [3–5], as well as the frequency of impulsive choices (for review, see [6, 7]). Further studies indicated that the effects of CBD on substance use are based on the ability of CBD to modulate the activation of dopaminergic brain circuits – including the ventral tegmental area (VTA) and nucleus accumbens (NAc) – that are closely linked to drug craving and drug-seeking [3, 8]. The potential of CBD for the treatment of substance use disorders was supported by two randomized placebo-controlled trials (RCTs) in patients with opioid use disorder (OUD) that showed significant craving-reducing effects of CBD in dosages of 400 and 800 mg daily, already a few hours after the first administration [8, 9]. In addition, an observational study in 120 alcohol and cannabis-using adults showed that ad libitum use of CBD-dominant cannabis over 5 days resulted in lower reported drinking days and drinks per drinking day compared to THC-dominant or THC/CBD-balanced cannabis [10]. Another RCT in individuals with cannabis use disorder showed higher abstinence rates from cannabis in individuals receiving 400 mg and 800 mg oral CBD daily versus placebo, supporting CBD’s potential for the treatment of substance use disorders [11]. For AUD however, clinical evidence is still lacking. Hence, we conducted the randomized, placebo-controlled ICONIC trial (“*Investigation of the effects of Cannabidiol ON cue-InduCed alcohol craving and nucleus accumbens activation*”) to determine the effects of CBD on stress- and cue-induced alcohol craving and NAc activation in individuals with AUD. Effects of CBD on craving were examined using a validated experimental stress- and cue exposure procedure [12, 13]. The effect of CBD on alcohol cue-induced NAc activation was tested using a validated alcohol cue-reactivity functional magnetic resonance imaging (fMRI) paradigm [14, 15]. We focused on the NAc as region of interest, because it was identified as a key neurobiological substrate of the addiction circuit [16, 17], and because neuroimaging studies demonstrated robust effects of alcohol cue presentation on NAc activation [18] and significant associations of NAc activation, alcohol craving [18] and relapse risk in AUD [19]. In addition, it was demonstrated, that higher activation in the ventral striatum is associated with the efficacy of naltrexone, supporting the potential of cue-induced brain response as a marker for individual efficacy of pharmacotherapeutic approaches [20]. The trial was designed to test the primary hypotheses that CBD reduces (i) cue-induced alcohol craving and (ii) NAc activation in individuals with AUD.

## Methods and materials

### Study design and procedures

This double-blind RCT was conducted at the Central Institute of Mental Health in Mannheim, Germany. The trial was approved by the local ethics committee (2022-579-AF 5), preregistered (German clinical trials database: DRKS00029993), and conducted in accordance to the Declaration of Helsinki. Enrolled participants were randomized to one of two groups (CBD, PLC) and completed one test session, which was scheduled on one day. On this day, individuals received CBD or PLC 3 hours prior to blood sampling for determining CBD levels, which was followed by a sequential stress- and cue exposure and an fMRI-based assessment of alcohol cue-induced NAc activation. The study was designed to detect at least medium effects (f ≥ 0.25) of CBD on the primary outcomes with a power of at least 80% (see Supplements for details).

### Participant recruitment

Non-treatment-seeking individuals with mild to severe AUD, between 18 and 60 years of age were recruited through online, social media, and newsletter advertisements. Potential participants were screened in a brief telephone interview and those who met preliminary criteria were scheduled for an on-site visit, where written informed consent was obtained and individuals were screened to determine their study eligibility. Specifically, the Structured Clinical Interview for DSM-5 was used to confirm the diagnosis of an AUD and to rule out any other substance use disorder (except tobacco use disorder) and severe psychiatric conditions (see Supplements for detailed exclusion criteria). Enrolled participants were randomly assigned to receive 800 mg of CBD or matching placebo. The block randomization schedule was produced by the independent study pharmacy and blinding was maintained until the last participant was assessed.

### Study drug

The oral CBD capsules (200 mg/capsule, consisting of >99.8% pure, synthetic CBD in a Hydroxypropylmethylcellulose capsule, THC content was <0.1%) and the matched oral placebo capsules were provided by Endosane Pharmaceuticals (Berlin, Germany). Packaging, blinding, and quality assurance were performed by the independent study pharmacy of Heidelberg University Hospital (Heidelberg, Germany). The CBD formulation was favored over other CBD products, because it does not contain ethanol (e.g., Epidiolex) and because regulatory agencies already approved its use in human trials. The dose was chosen in accordance to previous trials, which indicated a dose-response association with higher efficacy of a dosage of 800 mg CBD compared to lower dosages on drug craving [7, 8, 21]. The dose corresponded to an average weight-adjusted dose of 9.7 (range: 6.3–12.7) mg/kg CBD. The oral placebo capsules were identical in appearance, taste, and composition except for the active ingredient CBD. CBD or placebo (4 capsules) were administered once during the test session, 3 h prior to the combined stress- and cue exposure and the following fMRI. This schedule was chosen because previous work demonstrated peak plasma and brain concentrations of CBD 3–6 h after oral administration [22, 23].

### Procedure

**Fig. 1:**
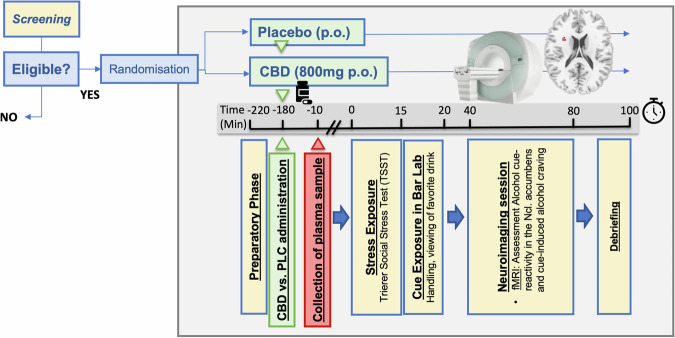
Schematic overview of the trial design. Either cannabidiol (CBD) or placebo (PLC) was administered after a preparatory phase at the beginning of the test session, followed by a rest period. 170 min after medication administration, venous blood was drawn for determination of CBD levels, and a combined stress- and cue exposure was conducted from minute 220–240 (i.e., 180 min after medication administration) using a combination of the Trier Social Stress Test (41) and an alcohol cue exposure in a bar lab setting (for details see text). Directly afterward, an alcohol cue-reactivity functional magnetic resonance imaging task was performed to investigate cue-induced brain activation and craving.

The timeline and design of the experimental session are depicted in Fig. 1. At the beginning of the session. All participants were instructed to remain abstinent for at least 24 h before starting the experimental session. They were screened for alcohol intoxication by determining breath alcohol concentration and drug use by drug urine screening and withdrawn if they tested positive. Women were additionally screened for pregnancy using urine pregnancy tests. Alcohol craving was assessed using the Alcohol Urge Questionnaire (AUQ) [24] and further questionnaires were administered to assess alcohol use prior to enrolment, AUD severity, nicotine use, stress, positive and negative effect, symptoms of depression and anxiety (see Supplements for details). Either CBD or PLC was administered at the beginning of the test session, followed by a rest period. After 170 min, venous blood was drawn for determination of CBD levels, and a combined stress- and cue exposure was conducted from minute 180–200 after medication administration using a combination of the Trier Social Stress Test (TSST) [25] and an alcohol cue exposure in a bar laboratory setting, which has been established and validated in previous studies as an experimental intervention for the induction of alcohol craving [12, 13] (for details see Supplements). Following the experimental craving induction, participants were transferred to the fMRI scanner where neural response to alcohol cues and craving were assessed, along with structural MRI (see Supplements). After that participants were debriefed on the TSST, i.e., that their performance during the task was not recorded and/or evaluated. In addition, well-being was assessed by the study personnel, and any side effects were noted.

### Primary and secondary endpoints

Alcohol cue-induced brain response in the NAc, measured using the blood oxygenated level dependent (BOLD) response, during presentation of alcohol cues, was preregistered as the primary outcome of the study. In addition, alcohol craving before and after the experimental combined stress- and cue exposure, measured using the AUQ, and cue-induced alcohol craving during fMRI, measured using a visual analog scale (0 – “no craving” to 100 – “very intense craving”), served as secondary study outcomes together with CBD plasma levels.

### Data analysis

The primary analysis included all randomized patients with fMRI data (N = 25). The primary endpoint was compared between both treatment arms using a t-test for independent samples (two-tailed), as implemented in the statistical parametric mapping software (SPM, Wellcome Department of Cognitive Neurology, London, UK) version 12 for Matlab (version 2016b, The MathWorks Inc., Natick, Massachusetts, USA), considering the first-level statistical maps that contrast activation during alcohol versus neutral blocks (contrast: “alcohol – neutral”) as input. Significance was set to a cluster-level family-wise error rate correction (FWE) of *p* < 0.05, considering the right and left NAc as pre-specified regions of interest (ROI), defined using an ROI mask from the NeuroVault repository (http://neurovault.org/media/images/12980/MNI_res-epi_label-NAcc_mask.nii.gz, see Supplementary Fig. S1). We performed additional sensitivity analyses considering days since the last alcohol use as a covariate in the model. To complement the analyses of averaged cue-induced brain activation, we also extracted block-wise activation values from the left and right NAc (see Supplements for details) and tested the main effect of the treatment group (k = 2 groups), time (i = 12 alcohol blocks) and the interaction between treatment x time in the framework of a General Linear Model (GLM) in the IBM Statistical Package for the Social Sciences (SPSS) version 29.0. Betas of alcohol and neutral blocks were compared using t-tests for independent samples in SPSS to test the specificity of the treatment effect. Alcohol craving was compared between treatment arms using GLMs in SPSS with treatment group (k = 2 groups) and time (i = 2 before/after combined stress- and cue exposure) for the AUQ score and time (i = 12 block-wise alcohol craving during fMRI) for alcohol craving during the fMRI session respectively. CBD levels were compared between treatment arms using t-test for independent samples. Associations between primary and secondary endpoints were explored using Pearson bivariate correlations in SPSS. Significance was set to p < 0.05 and corrected for multiple comparisons using the false discovery rate correction procedure and bootstrapping, using the Bias corrected and accelerated (BCa) bootstrapping procedure. Even though both treatment groups did not differ on any sociodemographic variable and there was no evidence for a significant impact of these variables in CBD levels, we performed additional sensitivity analyses considering sex (male/female) and current smoking status (yes/no) as covariates in the models. The inclusion of smoking status as a covariate essentially also captured the individuals reporting recent CBD or THC use.

## Results

A total of 28 individuals with AUD were enrolled in the study of whom 25 provided fMRI data for analyses of the primary endpoint (n_CBD_group_ = 12, n_PLC_group_ = 13; see Supplementary Fig. S2). For the secondary outcomes, data was available for all participants. Participants were on average 35.8 (*SD* = 12.1) years old, met an average of 5.4 (*SD* = 2.2) AUD criteria (18% mild, 46% moderate, 36% severe AUD), and drank on average of 46 g alcohol per day with an average of 36% heavy drinking days during the 90 days prior to the assessment. Prior CBD use was reported by 40% (lifetime) and 11% (last 3 months), and prior THC use was reported by 89% (lifetime) and 18% (last 3 months) respectively, but none of the participants showed a positive drug urine screening at the time of assessment. There were no significant differences between the treatment groups on any sociodemographic or substance use variable, neither when considering the whole sample (*N* = 28), nor for the groups contributing to the fMRI analyses (*N* = 25) (see Table 1 and Supplementary Table S1).

**Table 1:** Baseline data on demographic characteristics, alcohol use, and severity measures for participants randomized to the cannabidiol and placebo treatment arms.

	1	2		
	CBD (n = 14)^a^	PLC (n = 14)^a^	Statistics	Significance
Demographical variables (self-reported)				
Sex^b^ [male/female; number (%)]	11 (78%)/3 (22%)	8 (57%)/6 (43%)	Z = 1.47	*p* = 0.42
Age [years; mean (SD)]	37.29 (7.14)	34.36 (15.86)	*t*(18.06) = 0.63	*p* = 0.54
Race/ethnicity				
White [number (%)]	14 (100%)	14 (100%)	-	*-*
European ancestry [number (%)]	14 (100%)	14 (100%)	-	*-*
Substance use				
AUD criteria [sum; mean (SD)]	5.14 (2.21)	5.57 (2.24)	*t*(26) = 0.51	*p* = 0.62
Audit [total score; mean (SD)]	7.29 (1.68) 7	7.14 (2.18)	t(26) = 0.19	p = 0.85
ADS [total score; mean (SD)]	16.29 (7.60)	12.57 (4.80)	*t*(26) = −1.55	*p* = 0.13
Mean daily alcohol use last 90 days [gram/day; mean (SD)]	42.50 (35.04)	49.58 (29.57)	*t*(26) = 0.58	*p* = 0.57
Percent heavy drinking days last 90 days [mean (SD)]	0.30 (.24)	0.44 (.31)	*t*(26) = 1.32	*p* = 0.20
Days since last alcohol use [mean (SD)]	3.50 (4.18)	1−8, 2.71 (1.73)	*t*(26) = −0.65	*p* = 0.52
CBD use lifetime [yes/no; number (%)]	5 (36%)/9 (64%)	6 (43%)/8 (57%)	Z = 0.15	*p* = 1.00
CBD use last 30 days [yes/no; number (%)]	1 (7%)/13 (93%)	2 (14%)/12 (86%)	Z = 0.37	*p* = 1.00
THC use lifetime [yes/no; number (%)]	12 (86%)/2 (14%)	13 (93%)/1 (7%)	Z = 0.37	*p* = 1.00
THC use last 30 days [yes/no; number (%)]	4 (29%)/10 (71%)	1 (7%)/13 (93%)	Z = 2.19	*p* = 0.33
Current cigarette smoker [yes/no; number (%)]	3 (22%)/11 (78%)	3 (22%)/11 (78%)	-	*-*
Psychometric data				
AUQ at baseline (T0) [total score; mean (SD)]	15.36 (5.18)	15.93 (6.03)	*t*(26) = 0.27	*p* = 0.79
BDI [total score; mean (SD)]	13.21 (7.96)	18.71 (11.98)	t(26) = −1.43	p = 0.16
STAI trait [total score; mean (SD)]	43.14 (9.24)	50.57 (10.38)	t(26) = 2.0	p = 0.06
PANAS positive affect [total score; mean (SD)]	32.21 (7.57)	31.07 (5.41)	t(26) = −0.46	p = 0.65
PANAS negative affect [total score; mean (SD)]	25.43 (11.10)	26.00 (9.55)	t(26) = −0.15	p = 0.89
PASA [stress index score; mean (SD)]	−2.02 (1.03)	−1.79 (1.61)	*t*(22^b^) = −0.44	p = 0.67
CBD level (ng/ml)	256.76 (242.36)	0.42 (0.28)	*t*(13^c^) = −3.96	*p* = 0.002

*AUD* Alcohol Use Disorder, *AUDIT* Alcohol Use Disorders Identification Test, *ADS* Alcohol Dependence Scale, *AUQ* Alcohol Urge Questionnaire, *BDI* Beck Depression Inventory, *STAI* State Trait Anxiety Inventory, *PANAS* Positive and Negative Affect Schedule, *PASA* Primary Appraisal Secondary Appraisal.

^a^Remaining sample size for fMRI analysis is n = 12 for the CBD and n = 13 for the PLC group.

^b^Gender and sex were recorded by self-report and were consistent with each other.

^c^adjusted degrees of freedom according to standard procedures implemented in IBM SPSS version 29.0, due to unequal variances, indicated by positive Levene test.

### Cue-induced nucleus accumbens activation

**Fig. 2:**
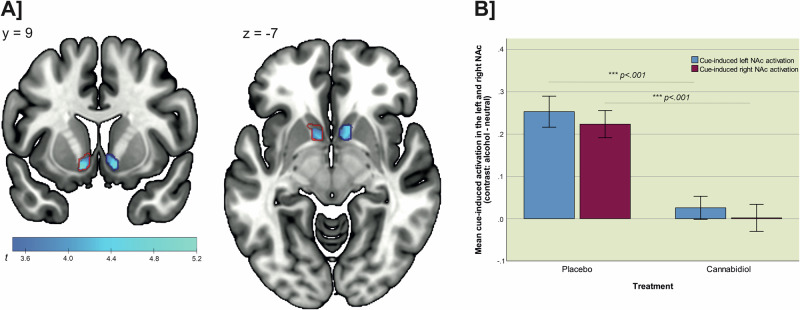
Treatment effect on cue-induced activation in the left and right NAc. Depiction of **A** the activation clusters in the left and right nucleus accumbens (NAc), which showed a significant treatment effect with significantly lower cue-induced activation in the CBD group (n = 12) compared to the PLC group (n = 13) (*p*_FWE_ < 0.05 small volume corrected for the NAc as pre-specified region of interest; boundaries of the region of interest mask are plotted as red and blue lines) and **B** the significant difference in mean alcohol cue-induced brain activation in the left and right NAc between the CBD and PLC groups (as part of a post hoc test, mean activation for the contrast “alcohol – neutral” was extracted using the pre-specified region of interest mask for the NAc and the marsbar toolbox for Matlab).

**Fig. 3:**
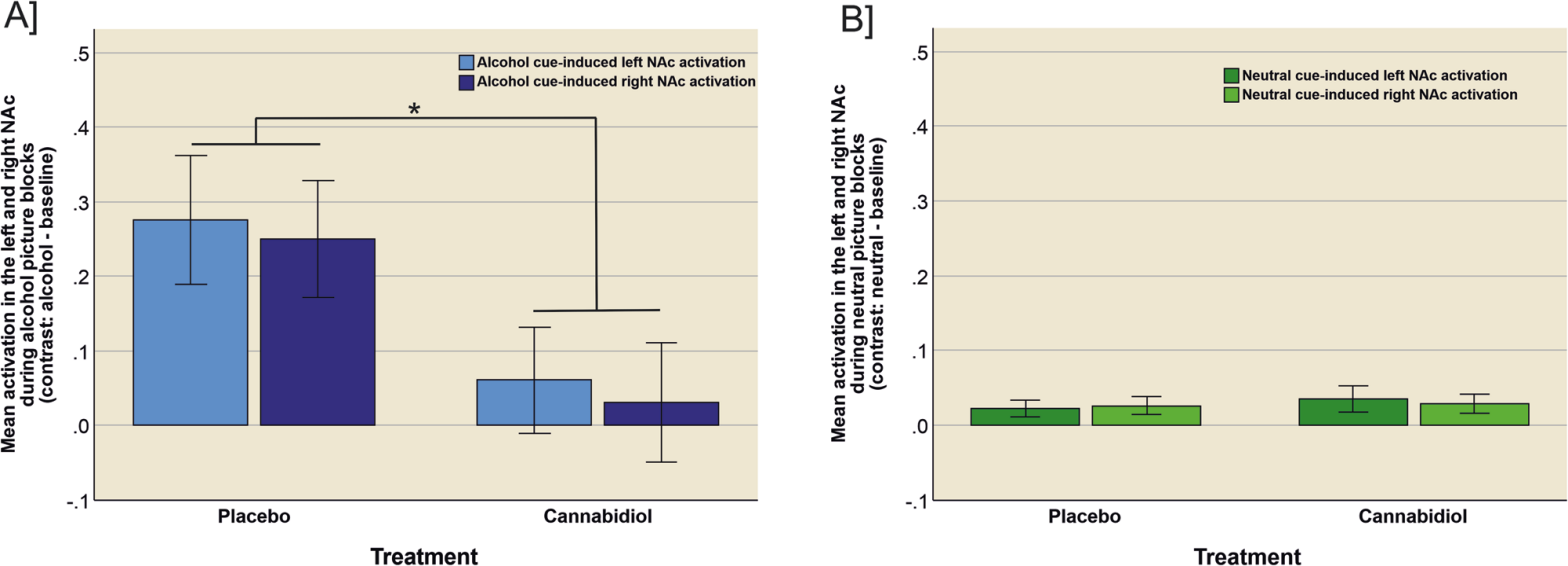
Cue-induced activation in the left and right NAc during alcohol and neutral stimuli. Depiction of the mean cue-induced activation in the left and right NAc during presentation of **A** alcohol stimuli (*t*_left NAc (23)_ = 4.150, *p* < 0.001, mean difference score = 2.150, d = 1.661, *t*_right NAc (23)_ = 4.281_,_ *p* < 0.001, mean difference score = 2.195, d = 1.714) and **B** neutral stimuli (*t*_left NAc (23)_ = −1.355, *p* = 0.188, mean difference score = −0.12, d = −0.543, *t*_right NAc (23)_ = −0.279, *p* = 0.783, mean difference score = −0.023, d = −0.112) for cannabidiol compared to placebo treatment.

Alcohol cues compared to neutral cues induced higher brain activation in several clusters, including parts of the occipital, temporal, parietal, and frontal cortices, the orbitofrontal cortex, as well as the NAc, cuneus, middle and posterior cingulum, precuneus, hippocampus, and the cerebellum (see Supplementary Table S2 and Supplementary Fig. S3). Individuals receiving CBD showed lower brain activation in the bilateral NAc compared to those receiving placebo (*p*_FWE left NAc (small volume correction)_ = 0.001, *p*_FWE right NAc [small volume correction]_ = 0.002, see Fig. 2). The effect remained significant, when considering days since last alcohol use as covariate (*p*_FWE left NAc [small volume correction]_ = 0.004, *p*_FWE right NAc [small volume correction]_ = 0.003). Analyses of the extracted mean NAc activation for the contrast “alcohol – neutral” showed large effects of the pharmacological intervention on left (*t*_(23)_ = 4.906, *p* < 0.001, d = 1.15) and right NAc activation (*t*_(23)_ = 4.873, *p* < 0.001, d = 1.13, see Fig. 2). No effects of CBD on brain activation in other brain areas were observed, even when considering a lenient uncorrected whole-brain threshold of *p* < 0.001. Analyses of block-wise activation values for the twelve alcohol picture blocks corroborated the significant main effect of the treatment group on cue-induced brain activation during alcohol cue-presentation blocks in the left and right NAc (*F*_left NAc(1,23)_ = 3.573, *p* = 0.047, partial eta^2^ = 0.12; *F*_right NAc(1,23)_ = 4.862, *p* = 0.034, partial eta^2^ = 0.18). The effects remained significant when considering gender and smoking status in the models (see Supplementary Table S3). In contrast, the analyses of block-wise activation values for the eight neutral picture blocks showed no significant main effect of group on either left or right NAc activity during neutral picture blocks (*F*_left NAc(1,23)_ = 0.978, *p* = 0.333, partial eta^2^ = 0.041; *F*_right NAc(1,23)_ = 0.055, *p* = 0.816, partial eta^2^ = 0.002), suggesting that the observed effects of CBD were specific for the presentation of salient alcohol cues. Additional analysis of betas supports the specificity of CBD-effects in the left and right NAc for the presentation of alcohol cues (*t*_left NAc (23)_ = 4.150, *p* < .001, mean difference score = 2.150, d = 1.661, *t*_right NAc (23)_ = 4.281, *p* < 0.001, mean difference score = 2.195, d = 1.714), but not for neutral cues (*t*_left NAc (23)_ = −1.355, *p* = 0.188, mean difference score = −0.125, d = −0.543, *t*_right NAc (23)_ = −0.279, *p* = 0.783, mean difference score = −0.023, d = −0.112) (see Fig. 3). There was no significant main effect of time or interaction between group and time on the left and right NAc activation, neither for the presentation of alcohol pictures nor for neutral picture blocks (*p* > 0.05).

### Cue-induced alcohol craving

**Fig. 4:**
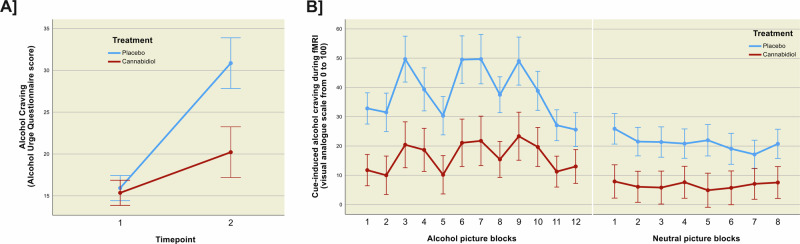
Treatment effect on alcohol craving. Depiction of the significant effects of cannabidiol **A** on alcohol craving before (*timepoint 1*) and after (*timepoint 2*) the combined experimental stress- and alcohol cue exposure (*F*_treatment_group(1,26)_ = 4.516, *p* = 0.043, eta^2^ = 0.15) and **B** on cue-induced alcohol craving during the fMRI cue-reactivity task (*F*_treatment_group(1,24)_ = 6.665, *p* = 0.015, eta^2^ = 0.23, n = 12 alcohol picture blocks and *F*_treatment_*_*_group(1,24)_ = 4.218, *p* = 0.052, n = 8 neutral picture blocks, errors bars represent to ±1 standard error).

At baseline, there was no significant difference between the groups regarding their AUQ craving scores (see Table 1). AUQ scores significantly increased from baseline to after combined stress- and alcohol cue exposure (mean difference score = 9.893, *F*_(1,26)_ = 21.798, *p* < 0.001, see Fig. 4). There was also a significant main effect of treatment group and interaction between treatment and time on AUQ scores (*F*_group(1,26)_ = 4.516, *p* = 0.043, eta^2^ = 0.15, mean difference score = 5.607; *F*_group x time(1,26)_ = 5.648, *p* = 0.025). Individuals receiving PLC reported a significantly greater increase in alcohol craving from before to after the combined stress- and alcohol cue exposure (mean difference score = 14.928) compared to participants receiving CBD (mean difference score = 4.857). Cue-induced craving during the fMRI alcohol cue exposure task was higher for alcohol compared to neutral blocks (*t*_(24)_ = 4.017, *p* < 0.001, mean difference score = 12.457, d = 0.803). There was also a significant main effect of the treatment group on craving during alcohol picture blocks (*F*_group(1,24)_ = 6.665, *p* = 0.015, eta^2^ = 0.23, mean difference score = 22.028, see Fig. 4). Individuals receiving PLC reported significantly higher alcohol craving during the cue-reactivity fMRI paradigm compared to participants receiving CBD. This effect remained significant when considering gender and smoking status in the models (see Supplementary Table S3). In contrast, there was no significant group effect on craving during neutral picture blocks (*F*_group(1,24)_ = 4.218, *p* = 0.052, mean difference score = 14.496).

### CBD plasma levels

Mean (*M*_CBD_ = 256.76 ng/ml, *M*_PLC_ = 0.41 ng/ml) and median (Med_CBD_ = 158.50 ng/ml, Med_PLC_ = 0.30 ng/ml) CBD plasma levels were significantly higher in the CBD group compared to the PLC group (*t*_(25)_ = 3.808, *p* < 0.001; U = 182, *p* < 0.001). Still, we observed substantial variance in CBD plasma levels (*SD* = 215.42 ng/ml), which mirrored the findings of previous studies (30). We explored whether age, gender, BMI, smoking status or recent CBD use (during the last 3 months) predicted CBD plasma levels, but neither of these factors showed a significant association with plasma levels (*p* > 0.05).

**Fig. 5:**
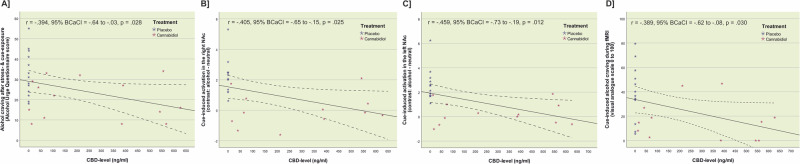
Correlation of CBD plasma levels with alcohol craving and NAc activation. Depiction of the significant negative correlation between CBD plasma levels and **A** alcohol craving after the combined stress- and alcohol cue exposure experiment (*r*_AUQ_ = −0.394, Bias corrected and accelerated 95% confidence interval [95% BCa CI] = −0.655 to −0.025, *p* = 0.028, *p*_FDR_ = 0.030), **B** alcohol cue-induced brain activation in the right nucleus accumbens (NAc) (*r*_right NAc_ = −0.405, 95% BCa CI = −0.642 to −0.161, *p* = 0.025, *p*_FDR_ = 0.030) and **C** left NAc (*r*_left NAc_ = −0.459, 95% BCa CI = −0.704 to −0.220, *p* = 0.012, *p*_FDR_ = 0.030), and **D** alcohol craving during the functional magnetic resonance imaging alcohol cue-reactivity task (*r*_craving fMRI_ = −0.389, 95% BCa CI = −0.624 to −0.075, *p* = 0.030, *p*_FDR_ = 0.030).

Testing associations between CBD levels and response on the primary and secondary outcomes, we found that CBD levels were negatively correlated with cue-induced left and right NAc activity during fMRI (*r*_left NAc_ = −0.459, Bias corrected and accelerated 95% confidence interval [95% BCa CI] = −0.704 to −0.220, *p* = 0.012, *p*_FDR_ = 0.030; *r*_right NAc_ = −0.405, 95% BCa CI = −0.642 to −0.161, p = 0.025, *p*_FDR_ = 0.030, see Fig. 5). Further, we observed a significant negative correlation between CBD levels and AUQ craving scores after the combined stress- and alcohol cue exposure session (*r*_AUQ_ = −0.394, 95% BCa CI = −0.655 to −0.025, *p* = 0.028, *p*_FDR_ = 0.030, see Fig. 5) and with alcohol cue-induced craving during the fMRI alcohol cue-reactivity task (*r*_craving fMRI_ = −0.389, 95% BCa CI = −0.624 to −0.075, *p* = 0.030, *p*_FDR_ = 0.030, see Fig. 5). These findings suggest a plasma level-response association for the primary and secondary outcomes.

### Safety

No adverse events or serious adverse events were reported by the participants of the study during the test session.

## Discussion

The results of this randomized double-blind placebo-controlled trial show that administration of 800 mg CBD reduces alcohol cue-induced bilateral NAc activity and alcohol craving in individuals with AUD. These results suggest that CBD can modulate central neurobiological mechanisms underlying alcohol craving and alcohol use and alleviate disease symptoms, such as craving. These effects were observed 3 h after acute administration of CBD, indicating rapid onset of CBD’s actions when peak plasma levels are expected [22]. These findings are in line with preclinical data [6] and also with a previous RCT in individuals with OUD that reported that the effects of CBD on drug cue-induced craving can be observed a few hours after administration of CBD [8]. Effects of CBD on alcohol use and relapse-like behavior in animals have been reported by several preclinical studies with bodyweight-adapted doses that fall in the range of the here investigated dose of 800 mg CBD [6, 7]. For the first time, the current RCT provides evidence for the significant effects of CBD on neurobiological disease mechanisms and symptoms in AUD. Effects of CBD on gene expression in the NAc were indicated by preclinical studies [4, 5], providing the basis for our focus on the NAc as region of interest. In addition, previous work showed close associations between NAc activity and AUD symptoms, such as alcohol craving [18, 26]. Further, our own group and others have repeatedly shown that the response of cue-induced NAc activation to pharmacological intervention is a predictor for the clinical efficacy of a drug for treating AUD [27, 28], stressing its relevance in investigation novel pharmacological interventions in AUD. Thus, the observed effects of CBD on cue-induced NAc activation, which was specific for the presentation of alcohol cues, indicate CBD’s potential to target central neurobiological disease mechanisms in AUD. Our findings are in line with previous RCTs in individuals at high risk for psychosis that showed significant effects of a single dose of 600 mg CBD on striatum activation during an fMRI verbal memory task [29] and processing of fearful faces during fMRI [30]. Further studies in individuals with psychosis also indicated that a single dose of 600 mg CBD, added to antipsychotic therapy, can significantly modulate mediotemporal-striatal connectivity [31]. The specificity of the effects of CBD and a plasma level-response association are supported by the significant correlation between CBD plasma levels and NAc activation. This is also in line with preclinical data indicating dose-dependent effects of CBD on alcohol use in animals [6] and evidence suggesting that plasma and brain levels of CBD are linked with about four hours to maximum brain concentrations after oral administration [23]. This corresponds closely to the schedule of the current RCT in which the effects of CBD on NAc activation were investigated about four hours after its administration.

CBD also showed significant effects on alcohol craving during an experimental combined stress- and cue-induction session and also during a following fMRI cue-reactivity task. The negative correlation between CBD levels and alcohol craving support the specificity and plasma level-dependency of the observed effects. CBD’s craving-reducing effects were specific to alcohol cue-induced craving, suggesting that CBD – via its effects on NAc activation – might attenuate alcohol-specific motivational salience in AUD and not appetitive behavior per se, leading to lower cue-induced “wanting” or craving [32]. This idea is in line with the finding that CBD attenuates attentional bias to cigarette cues in tobacco smokers, suggesting that CBD impacts on attentional salience of drug cues [33]. It also harmonizes with studies in individuals with psychosis, which showed that CBD attenuated the activation of salience networks during an fMRI monetary incentive delay task [34, 35]. CBD’s effects on craving appear clinically meaningful, as alcohol craving is a core symptom of AUD [36] and a potential marker for predicting the transition from moderate to severe AUD [37], emphasizing its role in AUD. Thus, pharmacotherapies targeting craving-related pathologies in AUD might be effective in reducing symptom burden and progression of the disease.

### Limitations

Several aspects of this RCT should be considered for the interpretation of the results. The RCT was designed to provide the first proof of CBD’s effects in AUD and thus investigated the effects of an acute single administration of CBD, as previous RCTs in other substance use disorders [8] and preclinical data indicated effects of CBD – if any –already after the first administration [6]. Presented results can thus not answer the question, whether CBD’s effect in AUD are robust over time, but preclinical [6] and clinical data in other substance use disorders [8, 11] indicate that effects are not transient and persist even after CBD is discontinued, suggesting that the here observed effects of CBD might translate to continued clinical effects in AUD. Higher activation during the presentation of the alcohol cues compared to neutral stimuli during the fMRI paradigm was evident in multiple brain regions, however CBD reduced alcohol cue-induced activity only in the NAc. This could be due to the small sample size. Besides that, the number of tests was also reduced to prevent from effects due to multiple testing. Both study groups were exposed to the same validated combined stress- and alcohol cue exposure paradigm [12, 13]. Thus, the effects of the intervention on the investigated outcomes are comparable between both study groups. However, it needs to be considered that stress- and cue exposure prior to the fMRI session mostly likely influenced outcome measures collected during fMRI. The AUQ score after the stress- and cue exposure session showed a significant difference between both study groups and we could not determine whether craving returned to baseline prior to the following fMRI cue-reactivity task. Thus, the here observed effects of CBD on neural cue-reactivity and craving during fMRI cannot be fully separated from effects on the prior stress- and cue exposure, i.e. the effects of CBD on cue-reactivity and cue-induced craving during the fMRI could be partially due to its effects on prior stress- or cue exposure or a combination of both. Hence, presented results cannot be generalized to cue-induced nucleus accumbens activation without prior stress- and cue exposure. Still, results seem meaningful as they inform about CBD’s effects on stress- and cue-reactivity in AUD and outcomes are comparable between both study arms as procedures were the same. We observed substantial inter-participant variability in CBD plasma levels, despite similar weight-adjusted doses across participants, which were not explained by either sex, age, BMI, smoking status or recent THC/CBD use, potentially owing to the complex metabolism and high lipophilicity of CBD, which was reported by preclinical studies [22, 23]. The here observed plasma level-response association for the primary and secondary outcomes of the study suggests that individuals might respond to the same CBD dose, highlighting the importance to consider CBD plasma levels and its determinants, when investigating CBD’s effects. The sample size of the presented RCT mirrored the sample sizes of previous RCTs [8, 11] and was based on a-priori calculations for the primary outcome, assuming that only at least medium effects of CBD on the primary outcome are clinically meaningful and warrant further larger confirmatory trials. These trials should also systematically investigate the effects of sex and determinants of individuals’ plasma CBD levels.

The current study provides evidence for the effects of CBD in non-treatment-seeking individuals with AUD. However, results might not generalize to treatment-seeking individuals with severe AUD. Further, the sample included predominantly white individuals with European ancestry, reflecting the national and European healthcare context and limiting heterogeneity. Still, results might not generalize to other populations, demanding for subsequent studies in these populations.

## Conclusion

In summary, the observed potential of CBD to reduce cue-induced NAc activity and alcohol craving, together with its good safety profile, supports the potential of CBD to treat individuals with AUD. New pharmacological treatment options that target central neurobiological disease mechanisms and core symptoms of AUD, such as craving, could complement existing treatment options and reduce relapse risk and the enormous disease burden inflicted by AUD.

## Supplementary information

Supplements

## Data Availability

The data that support the findings of this study are available on request from the corresponding author, SV. The data are not publicly available due to the sensitive nature of the data that could compromise the privacy of research participants.
