# Supplementary material for: Acute cannabidiol administration reduces alcohol craving and cue-induced nucleus accumbens activation in individuals with alcohol use disorder: the double-blind randomized controlled ICONIC trial
Source: Mol Psychiatry. 2024 Dec 12;30(6):2612–9. doi: 10.1038/s41380-024-02869-y (PMC12092268; doi:10.1038/s41380-024-02869-y)

SUPPLEMENTARY INFORMATION

Acute cannabidiol administration reduces alcohol craving and cue-induced nucleus accumbens activation in individuals with alcohol use disorder: the double-blind randomized controlled ICONIC trial

Zimmermann *et al.*

Content:

- **Supplementary Methods**
- **Supplementary Tables**
    - **Supplementary Table S1**. Baseline data on demographic characteristics, alcohol use and severity measures for participants randomized to the cannabidiol and placebo treatment arms with available neuroimaging data
    - **Supplementary Table S2**. List of brain areas that show a significant higher activation during the presentation of the alcohol cues compared to the presentation of neutral stimuli
    - **Supplementary Table S3.** Results of the sensitivity analyses for the primary outcomes.
- **Supplementary Figures**
    - **Supplementary Figure S1.** Depiction of the left nucleus accumbens region of interest mask
    - **Supplementary Figure S2.** CONSORT study flow chart
    - **Supplementary Figure S3.** Depiction of brain areas that show higher brain activation in response to the presentation of alcohol cues versus the presentation of neutral stimuli

Supplementary Methods

Sample Size Estimation

The study was designed to detect at least medium effects (f >= .25) of CBD on the primary outcomes with a power of at least 80%. This decision was taken, because the probability that smaller effects translate to clinically meaningful effects seems low. A priori sample sizes estimation indicated that *n* = 13 patients per group are needed to yield a power of at least 80% for a repeated measures analysis of variance with twelve repeated assessments testing the main effect of treatment group (f >= .25, α = 0.05, two-sided, correlation among repeated measures = .11, based on prior data [1]. Even though this number was not fully met for the imaging analyses (25 out of 28), power estimations still indicated a power of 81% to detecting at least medium effects (f >= .25, α = 0.05, two-sided, correlation among repeated measures = .10, based on current data).

Inclusion and Exclusion criteria

Participants were excluded when they tested positive for (i) alcohol (breath alcohol concentration [BrAC] > 0.0‰, alcohol consumption was not allowed in the last 24 hours before starting the test session) or (ii) illegal drugs (nal von minden - Drug-Screen Multi 5B test) or (iii) they presented withdrawal symptoms (Clinical Institute Withdrawal Assessment Scale > 3 points), or (iv) when DSM-5 interview indicated presence of a psychotic disorder, a bipolar disorder, or severe depressive symptoms with acute suicidality, or when they reported (v) pregnancy, lactation or breastfeeding, or (vi) any severe somatic comorbidities (e.g. liver cirrhosis or severely impaired renal function, severe heart insufficiency, pre-existing epilepsy), (vii) when contraindication for functional magnetic resonance imaging (e.g. metal implants) existed, or (viii) when participants reported hypersensitivity to Cannabidiol or to any excipient present in the pharmaceutical form of the investigational medicinal product.

Psychometric assessments and questionnaires

At the beginning of the testing session, participants were screened for excessive drinking using the Alcohol Use Disorders Identification Test (AUDIT) [2], severity of alcohol dependence was assessed

using the Alcohol Dependence Scale (ADS) [3], subjective stress was assessed using the Primary and Secondary Appraisal Scale (PASA) [4], positive and negative affect and depressive symptoms were assessed using the Positive and Negative Affect Schedule (PANAS) [5] and the Beck Depression Inventory (BDI-II) [6], alcohol use during the 90 days prior to enrolment was assessed using the Form-90 semi-structured interview [7], anxiety was assessed using the State Trait Anxiety Inventory (STAI) [8] , and nicotine use and nicotine dependence severity were assessed using the Fagerström Test for Nicotine Dependence (FTND) [9].

Combined stress- and alcohol cue-exposure session

The combined stress- and alcohol cue-exposure session was a combination of the Trier Social Stress Test (TSST) [10] and an alcohol cue-exposure, which has been established and validated in previous studies [1, 11]. The TSST is an established procedure for induction of psychosocial stress. The stressor includes a social evaluative part (public speaking in front of a panel) and a performance task (serial subtraction). Participants were informed that they will soon have an interview for their "dream job". They had five minutes to prepare a five-minute presentation. After this, participants entered a room, where a panel, consisting of two interviewers (male and female) that were unfamiliar to the participant, sat a table. These interviewers instructed the participant to give their presentation. During the presentation, the interviewers remained neutral, took notes and asked the participant to continue their presentation or give more information if they stopped for more than 20 seconds. After these five minutes, participants were instructed by the panel to perform a serial subtraction task for five minutes and received feedback on errors and were repeatedly told to perform subtractions quicker, even if performing well. Directly afterwards, participants entered a bar environment (Bar Lab), consisting of a mock-up bar, chairs and a bottle of their favorite drink positioned at the bar counter. In the Bar Lab, participants were exposed to their preferred drink. They were instructed to hold the drink in their hand for three to five minutes and smell it and swirl the glass, but without drinking any of it. Participants were alone in the Bar Lab, but were observed by clinical staff via a room monitor to ensure they were not drinking. The duration of the procedure was 20 minutes (15 minutes stress-exposure and 5 minutes

alcohol cue-exposure) and both groups underwent the same procedure to sensitize cue-reactivity before the fMRI examination.

Alcohol Cue-Reactivity Paradigm

Functional and structural MRI data was collected using a 3 Tesla SIEMENS Magnetom PRISMA^{FIT} MRI scanner (Siemens Healthcare, Erlangen, Germany). During the 12-minute alcohol cue reactivity paradigm (12), 840 T2*-weighted images (echo planar imaging sequence) were captured for each participant using the standardized image parameters (TR = 0.89 s, TE = 25 ms, flip angle = 80°, 42 slices, slice thickness = 2 mm, 1-mm gap, voxel dimensions 3 x 3 x 3 mm3, FOV = 192 x 192 mm2, 64 x 64 in-plane resolution). During the fMRI examination twelve blocks of alcohol-associated images and nine blocks of neutral images were presented (5 images per block) in a pseudo-randomized order using a screen and an MRI-compatible mirror. Each image was displayed for four seconds. Between each block, participants were asked to rate their current alcohol craving on a visual analog scale (VAS) ranging from 0 (no craving at all) to 100 (very intense craving). Image presentation and craving data collection were performed using Presentation® software (version 16.0, Neurobehavioral Systems Inc., Albany, CA, USA). The entire alcohol stimulus reactivity paradigm consists of 20 image blocks and takes twelve minutes to complete.

Processing and statistical modelling of functional MRI data

In order to reduce artefacts due to magnetic saturation effects, the first five scans were removed from subsequent processing. The remaining fMRI data was pre-processed using the statistical parametric mapping software (SPM, Wellcome Department of Cognitive Neurology, London, UK) version 12 and Matlab version 2016b (The MathWorks Inc., Natick, Massachusetts). A phase map correction was applied, in order to correct for any geometric distortions, using a voxel displacement map that was computed from a grey field mapping sequence using the VDM utility in SPM12. All images were spatially realigned, corrected for micro-movements in the scanner and normalized to a standard MNI [Montreal Neurological Institute, Quebec, Canada] EPI template. Subsequently, all data were smoothed using an isotropic Gaussian kernel for group analysis [8 mm Full Width at Half Maximum]). First level statistics were computed for each participant, modelling the different experimental

conditions (alcohol, neutral, rating phase) in a general linear model using the FAST method for temporal autocorrelation modelling [12], considering craving ratings and movements parameters (n=6) as nuisance variables. Resulting contrast images ("alcohol – neutral") were imputed in second-level analyses SPM12. In addition, following the approach established in previous studies [1, 13], we computed additional first level statistics, modelling each picture block of the fMRI cue-reactivity task separately (n=12 alcohol blocks, n=8 neutral blocks), in order to investigate block-wise brain activation. To this end, we used a custom toolbox to extract parameter estimates from individual beta maps for each stimulus block using the left and right nucleus accumbens mask from the Neurovault repository (http://neurovault.org/media/images/12980/MNI_res-epi_label-NAcc_mask.nii.gz) and exported these values into the IBM Statistical Package for the Social Sciences (SPSS) version 29.0 for further analyses.

Blood samples

Venous blood (plasma) was drawn 210 minutes after medication intake. Blood samples for analyses of cannabidiol levels were transported on ice and immediately transferred to the local biobank, where samples were centrifuged at 4000 rounds per minute (rpm) and plasma aliquots were stored at – 80°C and sent via express transport service in special cooling container for batch-analyses in a specialized laboratory (MVZ Labor Dessau GmbH, Dessau, Germany) to reduce inter-assay variation.

Chemical and reagents

Acetonitrile (ULC/MS-CC/SFC), methanol (ULC/MS-CC/SFC) and water (ULC/MS-CC/SFC) was purchased from Biosolve BV (Dieuze, France), ammonia solution (32%) and hydrochloric acid (37%) was from Merck KGaA (Darmstadt, Germany), formic acid (≥ 99.9%) from VWR (Leuven, Belgium), ethylene glycol (ROTIPURAN ≥ 99.5%) from Carl Roth (Karlsruhe, Germany), PBS tablets pH 7.2 (for 1 L) from AppliChem (Darmstadt, Germany). The serum quality control samples Medidrug® DOA-I S low and DOA-I S high were from Medichem (Steinenbronn, Germany) and a whole blood control sample STUP 07/20-B WH was from ACQ Science (Rottenburg-Hailfingen, Germany). The certified reference material cannabidiol (CBD) and the corresponding deuterated internal standard CBD-d3 was obtained from LGC Standards GmbH (Wesel, Germany).

Blood sample preparation

To 20 µL of plasma or control samples 200 µL MeOH/ACN (50/50, v/v) containing 2 ng/mL CBD-d3 was added. The mixture was vortexed and centrifuged at 13000 rpm for 5 min. Subsequently 55 µL of the supernatant was transferred to a 96-well plate and concentrated into 10 µL ethylene glycol at 60 °C in a vacuum evaporation centifuge. The residue was mixed with 15 µL of mobile phase A (20 mM ammonium formate, 0.1% formic acid) and 2 µL was injected into the UPLC system.

UPLC-MS/MS analysis

CBD was quantified using an DIN EN ISO 17025 accredited routine UPLC-MS/MS method. Data were acquired with a Waters® Acquity® UPLC® connected to a Xevo® TQ-XS detector with an UniSpray™ ion source (Waters®, Eschborn). The detector operated in the positive ionization mode. Chromatographic separation was performed at 60 °C on a Waters 2.1 mm x 150 mm, 1.7 µm, BEH Phenyl column with pre-filter. Mobile phase A consisted of 20 mM ammonium formate plus 0.1% formic acid (pH 3.0) while mobile phase B was 0.1% formic acid in methanol. Gradient separation was performed within 11 min at a flow rate of 0.3 mL/min. The gradient program initiated with 10% mobile phase B, increased to 100% at 10 minutes and remained for 0.5 minutes before re-equilibration. During data acquisition, 0.0037% HCl was infused post-column with a flow rate of 5 µL/min to enhance ionization. The autosampler temperature was maintained at 8 °C.

Compound-specific transitions monitored

	Retention time [min]	Parent-ions [m/z]	Daughter-ions [m/z]
CBD-d3	9.25	318.1	196.1, 262.1
CBD	9.25	315.1	193.1, 123.1, 259.1

Quantification was based on the response ratio of the target ion and the corresponding deuterated internal standard. Ten concentrations for CBD calibrators were prepared in EDTA whole blood ranging from 0.5 ng/mL to 120 ng/mL. The coefficient of determination (r2) was ≥ 0.99. Samples with concentrations exceeding 120 ng/mL were diluted 1:10 with PBS and re-analysed.

The method was validated following the GTFCh validation guidelines for quality assurance in forensic-toxicological analysis. Parameters evaluated include selectivity, linearity, limit of detection and quantification, precision, accuracy, matrix effect and stability. Full validation was performed using EDTA whole blood and the method was also cross-validated for plasma. Limit of detection (LOD) and limit of quantification (LOQ) were 0.3 ng/mL and 0.8 ng/mL, respectively.

References

1. Bach P, Zaiser J, Zimmermann S, Gessner T, Hoffmann S, Gerhardt S *et al.* Stress-Induced Sensitization of Insula Activation Predicts Alcohol Craving and Alcohol Use in Alcohol Use Disorder. *Biol Psychiatry* 2024; **95**(3)**:** 245-255.

2. Saunders JB, Aasland OG, Babor TF, De la Fuente JR, Grant M. Development of the alcohol use disorders identification test (AUDIT): WHO collaborative project on early detection of persons with harmful alcohol consumption-II. *Addiction* 1993; **88**(6)**:** 791-804.

3. Kivlahan DR, Sher KJ, Donovan DM. The Alcohol Dependence Scale: a validation study among inpatient alcoholics. *J Stud Alcohol* 1989; **50**(2)**:** 170-175.

4. Gaab J. PASA – Primary Appraisal Secondary Appraisal. *Verhaltenstherapie* 2009; **19:** 114-115.

5. Thompson ER. Development and validation of an internationally reliable short-form of the positive and negative affect schedule (PANAS). *Journal of cross-cultural psychology* 2007; **38**(2)**:** 227-242.

6. Hautzinger MK, F.; Kühner, C.; Beck, A.T. *Beck Depressions-Inventar : BDI II*. Frankfurt am Main : Pearson Assessment: Frankfurt am Main, 2009, 35 S. (Manual) + Fragebogenpp.

7. Miller WR. Form 90: A structured assessment interview for drinking and related behaviors: Test manual. 1996.

8. Spielberger C. *Manual for the State-Trait Anxiety Inventory*. Consulting Psychologists Press: Palo Alto (CA), 1983.

9. Heatherton TF, Kozlowski LT, Frecker RC, Fagerstrom KO. The Fagerstrom Test for Nicotine Dependence: a revision of the Fagerstrom Tolerance Questionnaire. *British journal of addiction* 1991; **86**(9)**:** 1119-1127.

10. Kirschbaum C, Pirke K-M, Hellhammer DH. The 'Trier Social Stress Test'–a tool for investigating psychobiological stress responses in a laboratory setting. *Neuropsychobiology* 1993; **28**(1-2)**:** 76-81.

11. Kwako LE, Schwandt ML, Sells JR, Ramchandani VA, Hommer DW, George DT *et al.* Methods for inducing alcohol craving in individuals with co-morbid alcohol dependence and posttraumatic stress disorder: behavioral and physiological outcomes. *Addict Biol* 2015; **20**(4)**:** 733-746.

12. Olszowy W, Aston J, Rua C, Williams GB. Accurate autocorrelation modeling substantially improves fMRI reliability. *Nature Communications* 2019; **10**(1)**:** 1220.

13. Gerhardt S, Berhe O, Moessnang C, Horning M, Kiefer F, Tost H *et al.* Lack of amygdala habituation to negative emotional faces in alcohol use disorder and the relation to adverse childhood experiences. *Addiction Biology* 2023; **28**(1)**:** e13251.

Supplementary Tables

Supplementary Table S1. Baseline data on demographic characteristics, alcohol use and severity measures for participants randomized to the cannabidiol and placebo treatment arms with available neuroimaging data (N=25).

	1	2		
	**CBD (n=12)**	**PLC (n=13)**	**Statistics**	**Significance**
Demographical variables (self-reported)				
Sex[A] [male/female; number (%)]	9 (75%)/3 (25%)	8 (62%)/5 (38%)	Z = 0.52	*p* = .67
Age [years; mean (SD)]	36.75 (7.38)	32.85 (15.43)	*t*(17.51) = 0.82	*p* = .43
Race/ethnicity				
White [number (%)]	12 (100%)	13 (100%)	-	-
European ancestry [number (%)]	12 (100%)	13 (100%)	-	-
Substance use				
AUD criteria [sum; mean (SD)]	4.75 (1.87)	5.38 (2.22)	*t*(23) = 0.77	*p* = .45
Audit [total score; mean (SD)]	7.33 (1.61)	7.00 (2.20)	*t*(23) = 0.43	*p* = .67
ADS [total score; mean (SD)]	15.33 (5.93)	12.38 (4.94)	*t*(23) = 1.36	*p* = .19
Mean daily alcohol use last 90 days [gram/day; mean (SD)]	37.06 (15.71)	48.39 (30.43)	*t*(23) = 1.15	*p* = .26
Percent heavy drinking days last 90 days [mean (SD)]	.27 (.14)	.41 (.29)	*t*(23) = 1.48	*p* = .15
Days since last alcohol use (mean (SD)]	3.75 (4.48)	2.77 (1.79)	*t*(23) = -0.73	*p* = .47
CBD use lifetime [yes/no; number (%)]	4 (33%) / 8 (67%)	6 (46%) / 7 (54%)	Z = 0.43	*p* = .69
CBD use last 30 days [yes/no; number (%)]	1 (8%) / 11 (92%)	2 (15%) / 11 (85%)	Z = 0.29	*p* = 1.00
THC use lifetime [yes/no; number (%)]	10 (83%) / 2 (17%)	12 (92%) / 1 (8%)	Z = 0.48	*p* = .59
THC use last 30 days [yes/no; number (%)]	3 (25%) / 9 (75%)	1 (8%) / 12 (92%)	Z = 1.39	*p* = .32
Current cigarette smoker [yes/no; number (%)]	3 (25%) / 9 (75%)	3 (23%)/10 (77%)	Z = 0.01	*p* = 1.00

Psychometric data[a]				
AUQ at baseline (T0) [total score; mean (SD)]	15.58 (5.45)	15.69 (6.21)	$t(23) = 0.46$	$p = .96$
BDI [total score; mean (SD)]	13.58 (8.21)	18.08 (12.22)	$t(23) = 1.07$	$p = .30$
STAI trait [total score; mean (SD)]	43.42 (8.48)	49.85 (10.43)	$t(23) = 1.68$	$p = .11$
PANAS positive affect [total score; mean (SD)]	32.17 (7.74)	25.85 (9.92)	$t(23) = 0.21$	$p = .84$
PANAS negative affect [total score; mean (SD)]	25.08 (10.98)	25.85 (9.92)	$t(23) = 0.18$	$p = .86$
PASA [stress index score; mean (SD)]	-2.18 (.99)	-1.94 (1.57)	$t(23^{B}) = 0.44$	$p = .66$
CBD level (ng/ml)	290.63 (245.31)	0.36(0.20)	$t(11^{B}) = -4.10$	$p = .002$

AUD = Alcohol Use Disorder, AUDIT = Alcohol Use Disorders Identification Test, ADS = Alcohol Dependence Scale, AUQ = Alcohol Urge Questionnaire, BDI = Beck Depression Inventory, STAI = State Trait Anxiety Inventory, PANAS = Positive and Negative Affect Schedule, PASA = Primary Appraisal Secondary Appraisal;

[A] Gender and sex were recorded by self-report and were consistent with each other;

[B]adjusted degrees of freedom according to standard procedures implemented in IBM SPSS version 29.0, due to unequal variances, indicated by positive Levene test.

Supplementary Table S2. List of brain areas that show a significant higher activation during the presentation of the alcohol cues compared to the presentation of neutral stimuli during the alcohol cue-reactivity functional magnetic resonance imaging paradigm (One sample t-test in SPM12 with sex (male/female), current smoking status (yes/no) and days since last alcohol use as covariates, considering all N = 25 participants contrast "alcohol – neutral", all results cluster-level whole-brain corrected at p_{FWE} < .05 and p_{FWE} < .05 small volume corrected for the nucleus accumbens as pre-specified region of interest).

Side	Lobe	Brain areas	Cluster size (voxel)	MNI coordinates (x, y, z)			t_{max}
Alcohol > Neutral							
L	Occipital	Calcarine Gyrus, Fusiform Gyrus, Superior/Middle/Inferior Occipital Gyrus, Cerebellum	5651	-38	-88	-6	11.12
R	Occipital	Fusiform Gyrus, Superior/ Inferior Occipital Gyrus, Cuneus, Calcarine Gyrus	2458	32	-90	-6	10.69
L	Frontal	Superior/Middle Frontal Gyrus, Supplementary Motor Area	675	-22	30	64	7.75
R	Frontal	Inferior/ Middle/ Superior Frontal Gyrus,	1094	48	42	32	6.63
L & R		Middle/Posterior Cingulum, Precuneus, Hippocampus	1339	2	-32	34	5.95
R	Parietal	Angular Gyrus, Superior/Inferior Parietal Gyrus	495	38	-62	48	5.94
L	Parietal/ Occipital	Angular Gyrus, Inferior Parietal Gyrus, Middle Occipital Gyrus, Superior Parietal Gyrus	1020	-34	-60	46	5.85
R		Cerebellum	372	54	-64	46	5.59
L	Frontal	Middle/Inferior Frontal Gyrus orbital parts, Middle Frontal Gyrus, Precentral Gyrus	1187	-46	16	32	5.39
R		Cerebellum	351	38	-58	-20	5.29

L	Nucelus Accumbens#	71	-12	10	-12	5.52
R	Nucleus Accumbens#	56	12	14	-6	5.25

Note. MNI = Montreal Neurological Institute; t_{max} = maximum t-value; p_{FWE} <.05; # = Region of interest (ROI) based small volume corrected (SVC) analysis.

Supplementary Table S3. Sensitivity analyses – Repeated measures analysis of variance models (RM-ANOVAS) with alcohol picture blocks (k=12) as within subject factor and treatment group (i=2) as between-subject factor and gender (male/female) and current smoking status(yes/no) investigating the main effect of treatment group on **A]** cue-induced activation in the left nucleus accumbens (NAc), **B]** right nucleus accumbens, and **C]** cue-indcued alcohol craving during the fMRI alcohol cue-reactivity paradigm.

**A] left NAc activation**			
	$F_{(1,21)}$	Significance	Partial Eta^2
Constant	2,242	,149	,096
Treatment group	4,279	,050*	,169
Gender	1,854	,188	,081
Smoking status	,591	,451	,027
**B] right NAc activation**			
	$F_{(1,21)}$	Significance	Partial Eta^2
Constant	1,607	,219	,071
Treatment group	4,321	,049*	,171
Gender	1,637	,215	,072
Smoking status	,116	,736	,006
**C] Cue-indcued craving**			
	$F_{(1,20)}$	Significance	Partial Eta^2
Constant	5,389	,032	,221
Treatment group	7,748	,011*	,279
Gender	,259	,617	,013
Smoking status	2,209	,153	,099

Supplementary Figures

Supplementary Figure S1. Depiction of the left (red) and right (blue) nucleus accumbens region of interest mask obtained from (http://neurovault.org/media/images/12980/MNI_res-epi_label-NAcc_mask.nii.gz)

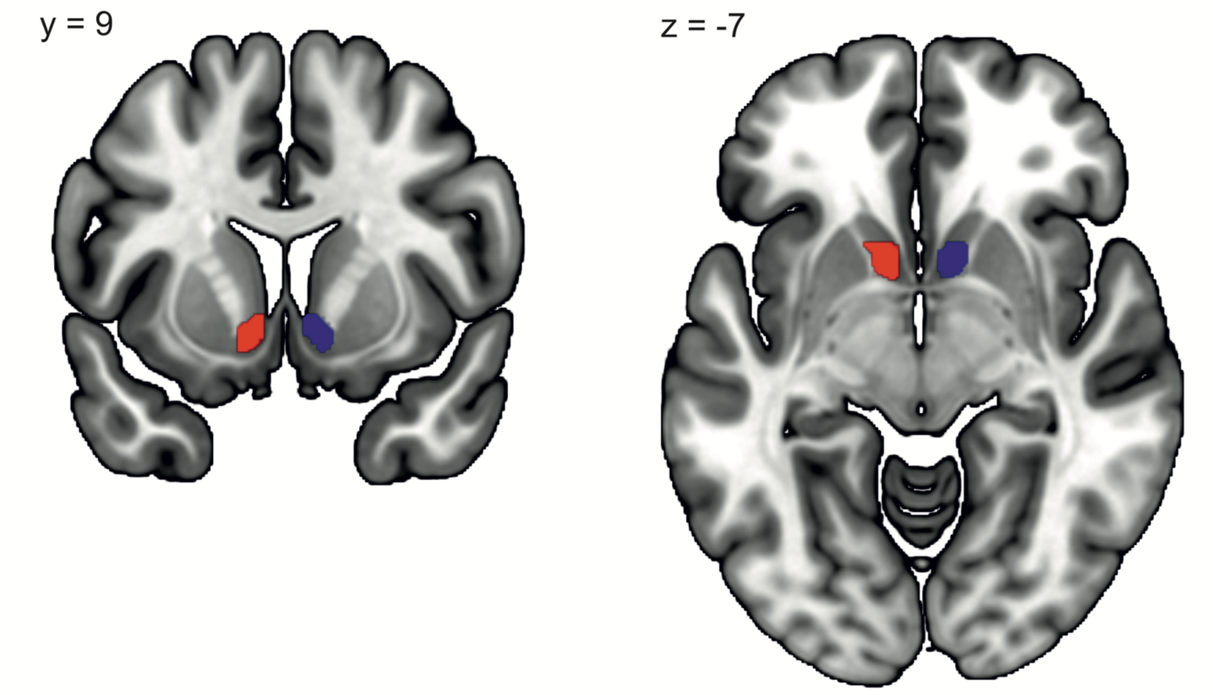

Supplementary Figure S2. CONSORT study flow chart

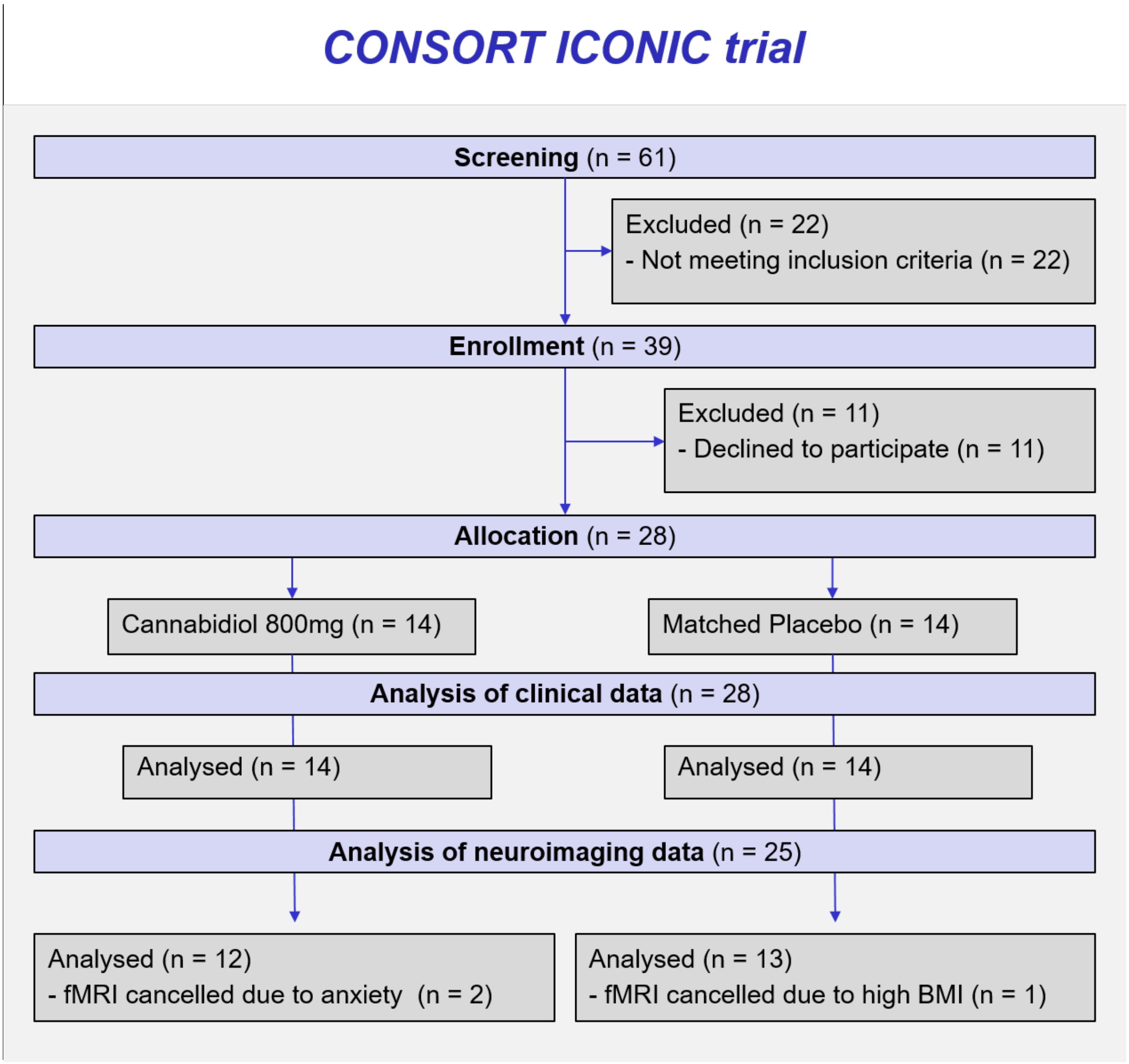

Supplementary Figure S3. Depiction of brain areas that show higher brain activation in response to the presentation of alcohol cues versus the presentation of neutral stimuli during the alcohol cue-reactivity task (One sample t-test with sex (male/female), current smoking status (yes/no) and days since last alcohol use as covariates in SPM12, considering all *n* = 25 participants contrast “aclohol – neutral”, all results cluster-level whole-brain corrected at $p_{FWE} < .05$ or $p_{FWE} < .05$ small volume corrected for the nucleus accumbens as pre-specified region of interest).

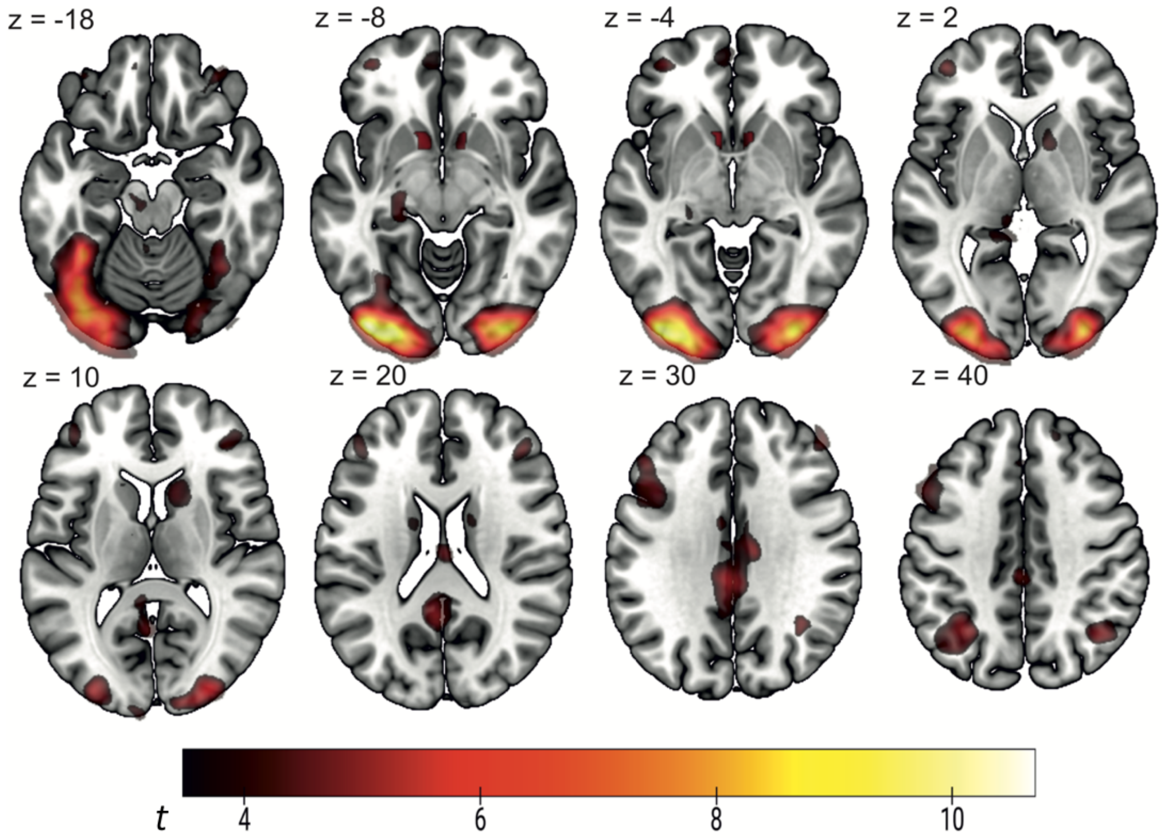

Supplement: Supplementary file 1 — Supplements [file 41380_2024_2869_MOESM1_ESM.pdf]
